# Comparative Study of Lightweight Target Detection Methods for Unmanned Aerial Vehicle-Based Road Distress Survey

**DOI:** 10.3390/s24186159

**Published:** 2024-09-23

**Authors:** Feifei Xu, Yan Wan, Zhipeng Ning, Hui Wang

**Affiliations:** 1 School of Civil and Transportation Engineering, Zhejiang Engineering Research Center of Digital Road Construction Technology, Ningbo University of Technology, Ningbo 315211, China; xufeifei@nbut.edu.cn (F.X.); wanyan@nbut.edu.cn (Y.W.); 2 Key Laboratory of New Technology for Construction of Cities in Mountain Area, Ministry of Education, School of Civil Engineering, Chongqing University, Chongqing 400045, China; localpn@outlook.com; 3 College of Artificial Intelligence, Southwest University, Chongqing 400715, China

**Keywords:** road distress survey, unmanned aerial vehicle, lightweight target detection, distributed shift convolution, deep separable convolution

## Abstract

Unmanned aerial vehicles (UAVs) are effective tools for identifying road anomalies with limited detection coverage due to the discrete spatial distribution of roads. Despite computational, storage, and transmission challenges, existing detection algorithms can be improved to support this task with robustness and efficiency. In this study, the K-means clustering algorithm was used to calculate the best prior anchor boxes; Faster R-CNN (region-based convolutional neural network), YOLOX-s (You Only Look Once version X-small), YOLOv5-s, YOLOv7-tiny, YOLO-MobileNet, and YOLO-RDD models were built based on image data collected by UAVs. YOLO-MobileNet has the most lightweight model but performed worst in accuracy, but greatly reduces detection accuracy. YOLO-RDD (road distress detection) performed best with a mean average precision (mAP) of 0.701 above the Intersection over Union (IoU) value of 0.5 and achieved relatively high accuracy in detecting all four types of distress. The YOLO-RDD model most successfully detected potholes with an *AP* of 0.790. Significant or severe distresses were better identified, and minor cracks were relatively poorly identified. The YOLO-RDD model achieved an 85% computational reduction compared to YOLOv7-tiny while maintaining high detection accuracy.

## 1. Introduction

Road distress detection is an important support for road infrastructure management. Existing research on road distress detection often focuses on a specific topic, including classification, detection, segmentation methods, and the solution of specific recognition problems, pursuing the high accuracy and generalization ability of the model. However, distress detection is often inseparable from specific engineering application scenarios. The requirements of detection equipment or measured target categories, and the detection accuracy corresponding to different work scenarios tend to be different [1,2], such as unmanned aerial vehicle (UAV) cruising [3], traffic recorder survey [4], and professional detection [5,6,7]. It has been noted that the shortcomings in the research of computer vision (CV) techniques for road distress detection include limited data quality and target categories [8,9,10].

Crack detection often focuses on accurate classification and segmentation. Some of the studies improve the robustness of crack segmentation by enriching the dataset [11,12], and some of these studies fall into the category of professional-level equipment detection using more expensive three-dimensional (3D) equipment [13,14,15,16]. Crack detection with higher accuracy and richer information has been realized based on 3D image data [13,14,15]. Huyan et al. proposed a state-of-the-art pixel-level crack detection architecture, CrackU-net, based on smartphone and digital camera images, which achieves pixel-level crack detection with acceptable precision [16]. However, the daily survey pays more attention to those problems that affect driving safety or the distress that needs to be repaired. With a daily repair management aim, Yang et al. [12] proposed a lightweight model that efficiently segmented seven types of road pavement distress targets based on front-view image data, and the influence of image quality on segmentation was discussed. Apeagyei et al. emphasized that the impact of data quality should be valued and studied [17]. Although the dominant algorithms are deep learning-based segmentation or target detection, some studies have also used classification algorithms as the core algorithm for distress detection. Hoang’s team has successively used machine learning classification algorithms to realize the classification of raveling and raveling degree [18], pothole repair and unrepaired potholes [19], cracking and sealing [20], aggregate segregation [21], and fatigue [22]. Lang et al. developed a multi-scale clustering algorithm based on 3D surface images to detect crack types [23]. Despite the high efficiency of these machine learning-based classification algorithms, their datasets are often produced with a certain bias, which is far from the actual survey application scenarios. The efficiency of the complete full-domain scanning detection and correlation algorithm was not analyzed. Specific unsupervised learning methods can still play a role in the preprocessing of data [24]. Hou et al. attempted a binary segmentation followed by a classification approach for crack detection [25]. Overall, due to the pixel-level processing of segmentation algorithms, which are often combined with specialized detection scenarios, the quality of the image data used is positively correlated with the segmentation effects [12,26,27]. Such algorithms have larger models, which are less computationally efficient and more difficult to combine with UAV applications.

Target detection algorithms in the deep learning category are more suitable for complex scenarios. Lei et al. proposed a deep learning method based on a pre-trained neural network architecture using Baidu map street view data based on YOLOv3 (You Only Look Once version 3) to achieve eight types of distress detection and verified that the model was able to effectively detect the generation and repair of distress based on three years of data from a specific roadway section [27]. The type and severity of cracks were detected using YOLOv5 based on specialized overhead images, and the medium-sized YOLOv5 performed the best [28]. Ning et al. proposed a YOLOv7-based effective detection model, YOLO-RDD (road distress detection), for eight types of pavement distress based on low-cost front-view video data; the accuracy and efficiency analyses of the model demonstrate its potential [4]. Wang et al. proposed an improved YOLOX-based two-step method for pavement pothole and loose detection, which shows significant advantages in terms of accuracy and efficiency [29]. Wu et al. developed a novel unsupervised domain adaptation framework and a self-supervised adversarial network for multi-category pavement distress classification on the unlabeled target domain, which performs well on specialized detection images [30]. Chen proposed a mobile integrated fifth-generation wireless communication technology (5G), cloud computing, and artificial intelligence (AI) automated system for the rapid identification of longitudinal cracks, transverse cracks, alligator cracks, and road-marking breaks [31], which is complete with testing a specific application scenario. Huyan implemented crack detection and measurement using multiple Gopro reconstructed 3D images to give a new way of detecting cracks at a low cost, but only analyzed crack detection and did not deal with other types of distress [32,33]. The YOLO family of algorithms resets the image to a uniform scaling size, where the size of the single image field of view determines the representation of the information.

In the study of efficient distress detection based on UAV, Zhu et al. built a pavement image dataset containing six types of pavement distress (transverse cracks, longitudinal cracks, alligator cracks, oblique cracks, potholes, and repairs), and YOLOv3 performed the best among the three algorithms (Faster R-CNN (region-based convolutional neural networks), YOLOv3, and YOLOv4) with a mean average precision (*mAP*) of 56.6% [34]. Zhang et al. achieved an *mAP* of 68.75% using UAV and YOLOv3 to detect longitudinal cracks, transverse cracks, repairs, and potholes [35]. Ma et al. achieved high accuracy and efficiency based on UAV-detected data, but only detecting cracks [36]. YOLOv7-tiny was reported in the leading position in terms of the number of model parameters, the amount of computation, and the accuracy, which proves the advancement of its network structure [37]. The algorithm data types used in related studies are summarized in Table 1.

It can be seen from Table 1 that few studies discussed the efficiency of the models, although they used the YOLO series of models. The positive impact of image quality on detection results has been declared by some studies [3,4,40]. However, it is more difficult to make side-by-side comparisons because the relationship between pixels and actual target size is not clearly expressed in most studies. UAV detection has a wide range of application scenarios with high-detection coverage and can be used to comprehensively survey problems in various road facilities, but it still faces great challenges in terms of cruising and data storage.

This study proposes to investigate two lightweight models for efficient road distress detection in UAV detection scenarios. Faster RCNN, YOLOX-s, YOLOv7-tiny, and YOLOv5s were used for the comparative study. Two lightweight convolutional operators were induced into the base model, namely depth-separable convolution and distribution-shift convolution aiming to be lightweight while improving its detection accuracy as much as possible for UAV detection.

## 2. Data and Methods

### 2.1. Dataset

The “China_Drone” data in RD22 (Road Distress Dataset in 2022) [43] was used as the dataset for the experiments in this paper. This dataset contains 2401 images with a resolution of 512 × 512, which are all derived from the road distress images of China captured by UAVs. In this study, a total of 1919 images were selected after data cleaning. The raw unprocessed images were divided according to a training–validation–test ratio of 1119:400:400.

**Figure 1:**
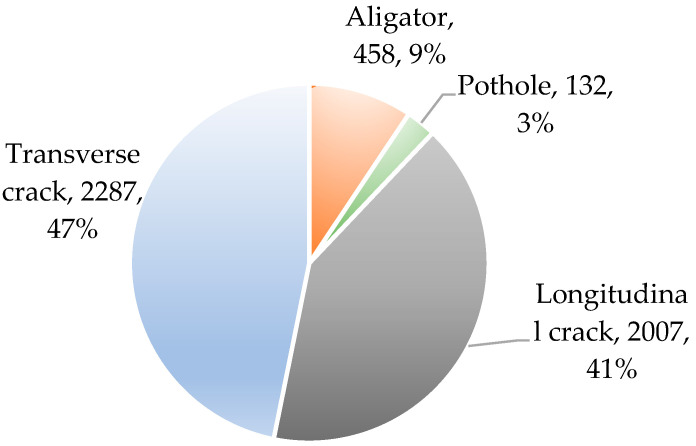
Data distribution.

**Figure 2:**
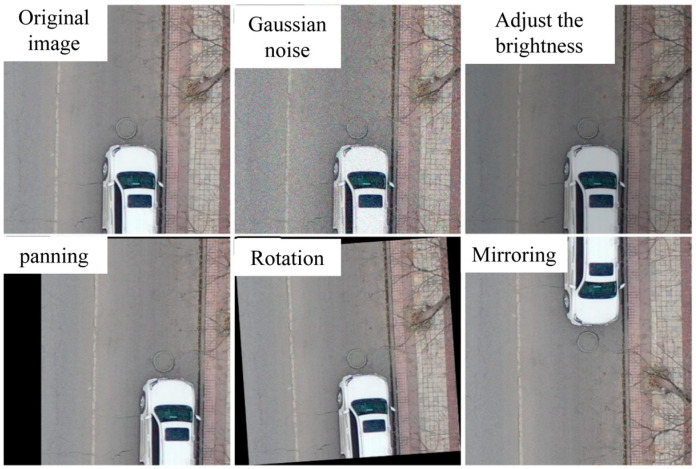
Data augmentation samples.

To address the problem of fewer training data samples, data augmentation in the form of Gaussian noise, adjusting brightness, and geometric transformations (translation, rotation, and mirroring) were performed on each image in the training set. The label distribution of the dataset after data augmentation is shown in Figure 1 and the augmentation representative samples are shown in Figure 2.

### 2.2. Methodology

#### 2.2.1. Priori Box Clustering

Priori box (anchor box) is a set of bounding boxes defined in advance at a fixed size and scale in the image. These priori boxes are used to sample regions in the image that may contain targets and provide the model with an initial guess of the location and size of the target. The a priori boxes are usually a set of rectangular boxes uniformly distributed on the feature map, each associated with a specific spatial location and scale. In the YOLO family of target detection networks, the anchor boxes are usually set as a set of predefined bounding boxes, the number of which is typically nine. During the training process, the YOLO model uses these priori boxes to predict possible targets in the image. By adjusting the offset and scale of each priori box, the model tries to make these priori boxes better match the position and size of the real target. By comparing with real labels, the model learns how to adjust these priori boxes so that the final detection box can be as close as possible to the real target.

Therefore, the choice of a priori frames is crucial to the performance of target detection. A suitable priori frame design can help the model detect targets of various sizes and proportions more effectively and can reduce the computational burden of the model during training. However, for different datasets, different a priori frame settings bring different results, so there is no a priori frame that is optimal for all datasets. For example, the a priori box used in Faster R-CNN is designed by the authors through their engineering experience, and no reason is given for such a design. To address these issues, this study uses the K-means clustering algorithm to calculate the best prior frame. The final cluster assignment result is obtained when the clustering center no longer changes.

#### 2.2.2. Backbone Improvement with MobileNetV3

Based on deeply separable convolution, MobileNetV3 [44] is used as the backbone network for feature extraction. A search space approach was used to design the network structures. This approach allows automatic search and evaluation of multiple combinations of operations and structures to find the optimal network structure. In addition, MobileNetV3 introduces a module called “Inverted Bottleneck” which combines linear bottleneck and nonlinear scaling operations. The proposed method uses a combination of techniques to optimize network performance. Specifically, low-dimensional convolution is used to reduce computation, while a lightweight activation function (h-swish) is incorporated to enhance the nonlinear capabilities of the network. The backbone network layer of YOLOv7-tiny is replaced by the MobileNetV3 network to obtain a road distress detection model with a smaller size and faster computation speed. 

The input image is adjusted to the size of 640 × 640 pixels in the input layer. The resized image passes through the CBHw module, which consists of the depth-separable convolution, the batch normalization layer, the Hard Wish activation function, and 11 feature extraction benck modules [45], as shown in Table 2. 

#### 2.2.3. Multi-Scale Feature Fusion Optimization

Convolutional neural networks capture a wider range of contextual information as the number of layers increases. Although this can lead to the loss or blurring of detailed features, shallow networks with smaller receptive fields are better at capturing local detailed features. To address these issues, a multi-scale feature fusion structure [46] has been induced as an effective solution. This structure merges the feature maps of shallow and deep networks to obtain a feature representation that contains both global and detailed information.

**Figure 3:**
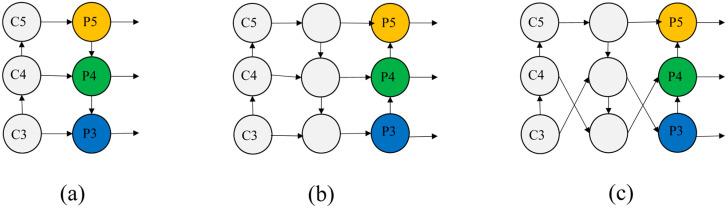
Feature fusion mode. (**a**) Top-down fusion; (**b**) simple bi-directional fusion; (**c**) complex bi-directional fusion.

Common feature fusion structures typically include top-down fusion, simple bi-directional fusion, and complex bi-directional fusion, as shown in Figure 3. C3, C4, and C5 represent the input feature maps, while P3, P4, and P5 represent the output feature maps. The most commonly used structure for multi-scale feature fusion is the top-down fusion approach, which is employed by common algorithms such as Faster R-CNN and YOLOv3. As the network deepens from C3 to C5, the output feature maps gradually become smaller but contain richer information after the pooling operation. The feature maps are then enlarged by upsampling at the P5 to P3 layers, ensuring that feature maps of different depths have the same size. This enables seamless multi-scale feature fusion. This is in contrast to simple bi-directional fusion, which only performs secondary fusion based on top-down fusion, combining the feature information at multiple levels. The model comprehensively understands the features and semantic information of the input data through complex bi-directional fusion, which considers both forward and reverse feature propagation, allowing for the interaction and fusion of information from different directions.

Simple bi-directional fusion is used in YOLOv7-tiny, where the output of the 14th convolutional layer is used as the C3 layer and the 21st convolutional layer is used as the C4 layer. This study employs a simple bi-directional fusion approach. The C3 layer is derived from the benck3 module output in the MobileNetV3 backbone network, while the C4 layer is derived from the benck8 module output. The corresponding layers of the YOLOv7-tiny head network undergo the same multi-scale feature fusion through concatenation.

#### 2.2.4. YOLO-MobileNet

The MobileNet used a depth-separable convolution, which is a lightweight convolution operator that performs a separation operation between the two parts of the convolution operation process, including depth-by-depth convolution and point-by-point convolution. In depth-by-depth convolution, a convolution kernel is responsible for only one channel, while a channel is convolved by only one convolution kernel. Point-by-point convolution is a weighted summation of the feature maps obtained in the previous step, and as many convolution kernels as possible will generate as many new feature maps as possible, ensuring the same dimensionality as the output of regular convolution. Its computation and resource occupancy are low, and the network structure is lightweight to a high degree.

**Figure 4:**
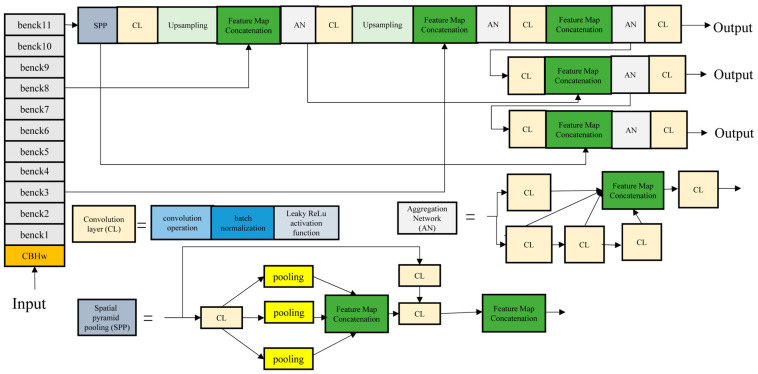
The whole framework of YOLO-MobileNetV3.

The whole network framework of YOLO-MobileNet is shown in Figure 4. 

The framework consists of two main components: the backbone network and the head network. The MobileNet structure is used in the backbone network to extract features from the input image. The head network consists of the SPPCSPC spatial pyramid module, multiple convolutional pooling, upsampling, and concat feature operations to convert the feature mapping into target detection results. The convolutional layers of the head network include depth-separable convolutions, batch normalization layers, and LeakyReLU activation functions. These modules extract critical image features, normalize them, and perform nonlinear transformations to effectively support the target detection task. The backbone and head network layers are bidirectionally connected through a multi-scale feature fusion approach. This enables the exchange and fusion of feature information between layers, synthesizing information from different depths and scales to improve detection performance and reduce model complexity. This approach uses bidirectional multi-scale feature fusion to improve the model’s understanding of the input image and produce accurate target detection results.

#### 2.2.5. YOLO-RDD

YOLO-RDD [4] has been chosen based on its considerable performance in accuracy and efficiency of front-view video-based detection. YOLO-RDD uses distribution-shift convolution [47] instead of ordinary convolution. Distribution-shift convolution decomposes the traditional convolution kernel into two components: the first component is an integer-only tensor with a composition of low integer values that have the same size as the original convolution tensor, called the variable quantization kernel. The other component has a fraction of the size of the original tensor, consisting of floating-point scaling factors, called the kernel distribution shifter, which maintains the relative distribution of weights and activation values over the kernel by scaling and shifting them. Such quantization allows the distribution-shifted convolution to operate with low-precision values during training and take up less memory while having faster inference and maintaining high-precision detection results.

**Figure 5:**
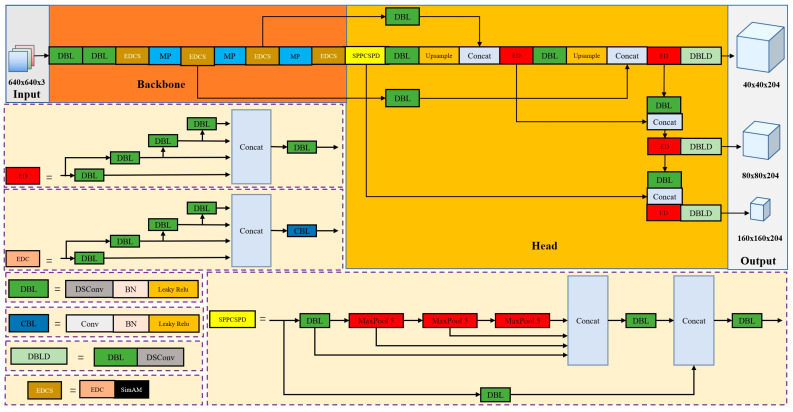
The framework of YOLOv7-RDD [4].

The framework is shown in Figure 5. In the backbone network of YOLO-RDD, the initial convolution consists of two convolutional layers, each consisting of a distribution-shift convolution, a bulk normalization layer, and a LeakyRelu activation function. This is followed by three sets of fusion modules and downsampling operations, where the fusion module is a SimAM attention mechanism incorporated in the tail of the EDC (efficient lightweight aggregation network with DSConv and Conv) structure of the aggregation network to enhance the characterization of the feature map. In the head network, the improved spatial pyramid pooling structure SPPCSPD is employed for enhancing the extraction of multi-scale features. Then, the size of the feature map is adjusted by the ED module (efficient lightweight aggregation network with DSConv) and the upsampling operation is performed for the fusion of multi-scale features with the backbone network layer. Finally, the output is performed after one convolution operation.

## 3. Experiment

### 3.1. Experimental Environment

The experimental environment of this paper is based on Windows 11, Python 3.10.9, the Pytorch ‘1.12.1+cu11.7’ framework, CUDA (Compute Unified Device Architecture) 11.7, CuDNN (CUDA Deep Neural Network library) v8.8, and the hardware facilities are Intel i5-12500 2.5G Hz, NVIDIA GeForce RTX 3060, and the implementation of the experiment mainly uses libraries such as CUDA, CuDNN, and OpenCV (Open Source Computer Vision Library). The generic parameter configuration is shown in Table 3.

### 3.2. Evaluation Metrics

In the detection model evaluation for UAV data, precision (*Precision*), recall (*Recall*), true negative rate (*TNR*), balanced accuracy (*BA*), mean average accuracy (*mAP*), and reconciled mean score (*F1-score*) are commonly used as the precision evaluation indicators, parameter number (*param*) and operation volume (*FLOPs*) are used as the evaluation indicators of the model size, which are calculated according to Formula (1) to (7).

(1) $$Precision=\frac{TP}{TP+FP}$$

(2) $$Recall=\frac{TP}{TP+FN}$$

(3) $$TNR=\frac{TN}{TN+FP}$$

(4) $$BA=\frac{Recall+TNR}{2}$$

(5) $$AP=\int_{0}^{1}PdR$$

(6) $$mAP=\frac{1}{N}\sum_{i=1}^{N}AP_{i}$$

(7) $$F1=2*\frac{Precision*Recall}{Precision+Recall}$$

where *TP* stands for true positive, *FP* stands for false positive, *TN* stands for true negative and *FN* stands for false negative. *AP* stands for the area enclosed by the PR curve with x and y coordinates, and *N* stands for the number of detection categories. 

## 4. Results and Discussion

### 4.1. K-Means Clustering Results

**Figure 6:**
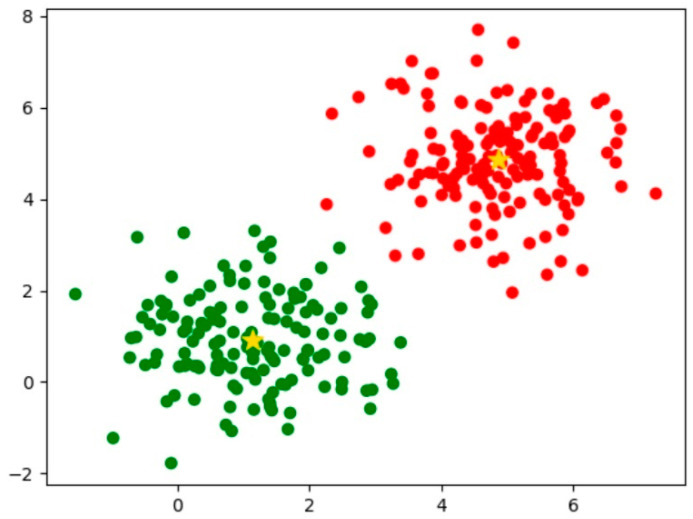
K-means clustering example.

In this paper, K-means clustering is performed for 1000 iterations for the labels corresponding to the 2238 images in the training set, and nine prior frames are finally obtained; the running results are shown in Figure 6. They are [84, 29], [34, 115], [178, 39], [34, 217], [55, 238], [282, 58], [59, 518], [462, 89], and [220, 220].

### 4.2. Comparison of Improved Models

**Figure 7:**
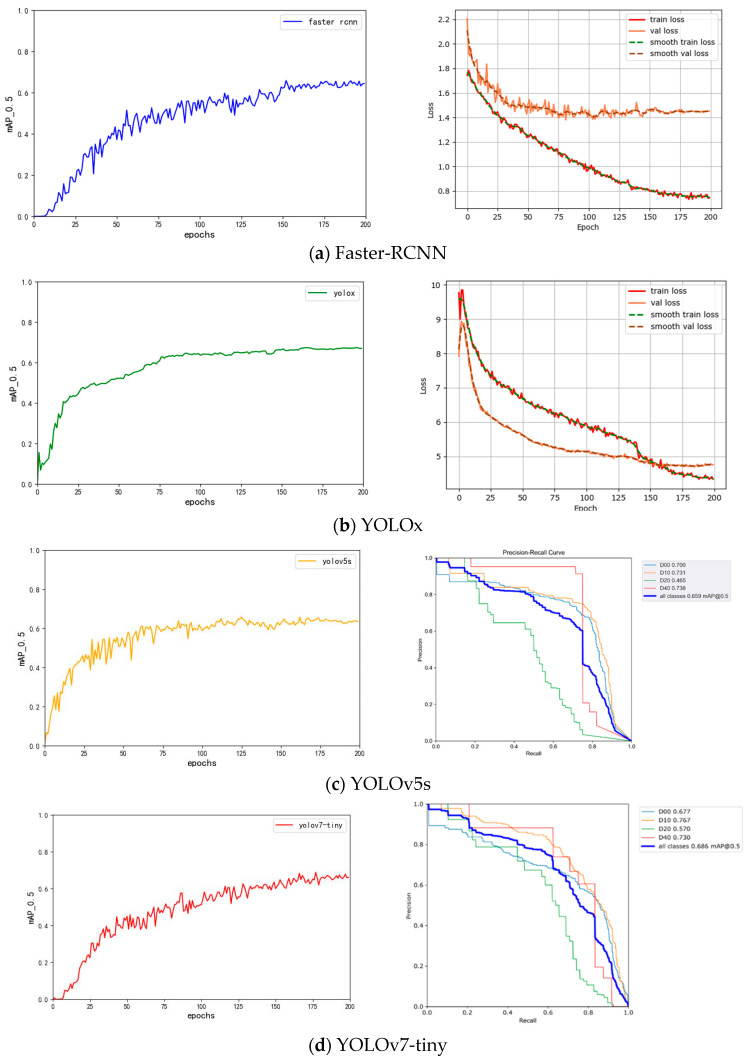
Train results of the comparative models. D00 represents longitudinal cracks, D10 represents transverse cracks, D20 represents net cracks, and D40 represents potholes.

The training process of each model is shown in Figure 7.

As can be seen from Figure 7, the accuracy of the Faste-RCNN model starts to converge when the training reaches 150 rounds, the model experiences good parameter tuning during the training process, and the detection accuracy is significantly improved, the loss on the validation set (val loss) starts to stabilize when the training reaches 125 rounds, and the decrease in the loss on the training set (train loss) decreases, which indicates that the training of the model is close to the optimal state and there is no need to add more training rounds. The YOLOX-s training process remains consistent with the change of accuracy, especially after 175 rounds, the average value of the loss in both training and validation is almost unchanged, which indicates that the model has reached a very good convergence situation. The convergence speed of the training phase of YOLOv5s is substantially improved compared to that of the Faster R- CNN; YOLOv5s shows a huge improvement in accuracy in the first 50 rounds of training, and the model corrects the prior frames faster in the early stage. The convergence of YOLOv5s slows down when the number of training rounds reaches 100, and at around 200 rounds, the accuracy hardly changes, and the training of the model has reached a very good convergence effect. YOLOv7-tiny does not converge as fast, and the accuracy starts to stabilize after 175 rounds. The YOLOv5s model reached an *mAP@0.5* of 0.659, slightly higher than Faster R-CNN, and slightly lower than YOLOX-s. YOLOv7-tiny’s overall accuracy is superior to that of Faster R-CNN, YOLOX-s, and YOLOv5s, and it is in the leading position. YOLOv7-tiny, as the smallest model of the YOLOv7 series, has fewer parameters, and less computation. 

The comparison results of the model proposed are shown in Table 4.

Table 4 shows that the YOLO-RDD model has the highest accuracy among the models compared, with an mAP@0.5 of 0.701. It is important to note that Faster R-CNN is not suitable for this application due to its large *param* and *FLOPs* values compared to the other six models. In addition, YOLOX-s did not outperform YOLOv7-tiny in all metrics and achieved only slightly higher accuracy results than YOLOv5s. The YOLO-RDD method efficiently computes the quantization and scaling in the distribution shift convolution, resulting in an 85% computational reduction compared to YOLOv7-tiny, while maintaining high detection accuracy. It is worth noting that although YOLO-MobileNetV3 reduces the number of parameters and computations by 25% and 48%, respectively, its accuracy is lower than YOLOv7-tiny, resulting in a mAP@0.5 of only 0.460. MobileNetV3 uses a linear convolutional process as its backbone network, which differs from the efficient aggregation of the YOLOv7-tiny network. However, the feature extraction phase could benefit from improved feature splicing, despite the use of a multi-scale feature fusion approach. 

**Figure 8:**
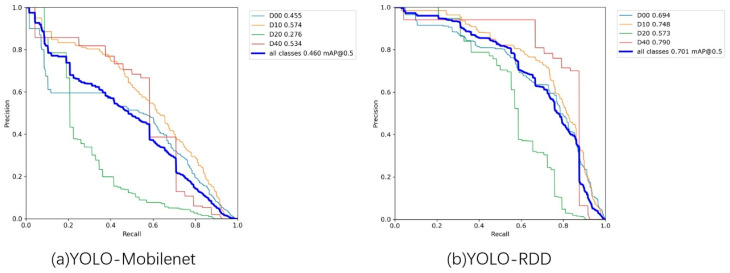
PR curves of YOLO-MobileNet and YOLO-RDD experiments. D00 represents longitudinal cracks, D10 represents transverse cracks, D20 represents net cracks, and D40 represents potholes.

The experimental PR curves of YOLO-RDD and YOLO-MobileNet are drawn in Figure 8 for further analysis.

Figure 8 demonstrates that YOLO-RDD achieves high accuracy in detecting all four types of distress, with the most successful detection being potholes (D40) with an *AP* of 0.790. Potholes are identified by their shape, whereas longitudinal cracks (D00), transverse cracks (D10), and net cracks (D20) are identified by their texture. The texture is a more intricate feature that can be challenging to detect, particularly in the case of net cracks, which have complex and irregular texture information, resulting in the lowest accuracy. The YOLO-RDD model effectively meets the requirements of practical applications by achieving high recognition rates for various types of distresses. In contrast, the YOLO-MobileNet model, despite having the smallest model complexity, performs poorly in terms of detection accuracy. Its detection accuracy for transverse cracks (D10) is only 0.574, while the detection accuracy for net cracks (D40) is even lower. For MobileNet, the operation of decomposing traditional convolution into deep convolution and point-by-point convolution drastically reduces the computational complexity and the number of parameters, lowering the computational cost and storage requirements, but also reducing the fusion interaction of information. The corresponding recognition accuracy is reduced. Distributed shift convolution outperformed deep separable convolution in the road distress detection task. 

### 4.3. Case Studies of Experimental Results

**Figure 9:**
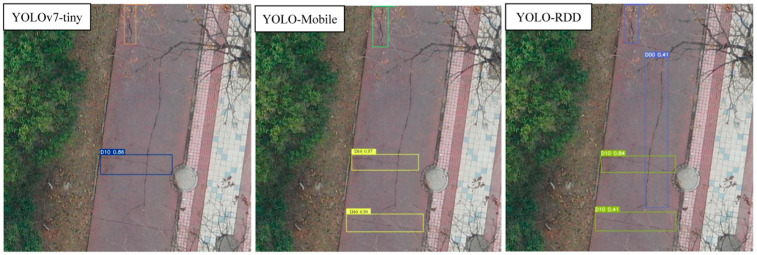
Crack detection results.

**Figure 10:**
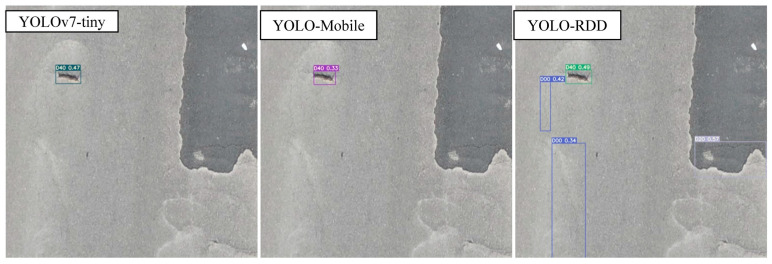
Pothole detection effect.

**Figure 11:**
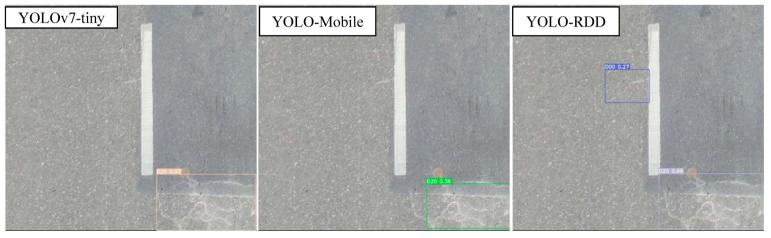
Net crack detection effect.

To compare the models more intuitively, this subsection conducts a visualization of the effect as shown in Figure 9, Figure 10 and Figure 11.

Figure 9 shows that YOLOv7-tiny detected one transverse crack (D10) and one longitudinal crack (D00), while YOLO-MobileNet additionally detected the transverse crack (D00) on top, and YOLO-RDD was the only model that could detect the longitudinal crack (D00) in the center of the figure, while also detecting the two transverse cracks (D10) mentioned above.

Figure 10 shows that all three models were able to detect the pothole in the center. YOLO-RDD was the only model that detected the longitudinal cracks (D00), which were missed by the other two models. It is worth noting that the one longitudinal crack (D00) and one net crack (D20) detected in the figure, are slight longitudinal depressions that are misidentified. This observation suggests that the models may be overfitting. As this is an FP problem, it can be solved by increasing the target types of distress and expanding the dataset.

In Figure 11, both YOLO-MobileNet and YOLOv7-tiny detected the net crack located in the lower right corner; insufficiently, the detection box of the YOLO-MobileNet model did not contain all of this target. Specifically, YOLO-RDD successfully detected both the net crack in the lower right corner and the transverse crack (D00) in the center of the figure, while YOLO-MobileNet only detected a portion of the net crack (D20). Overall, YOLO-RDD is the most effective model for this task. The results in Figure 10 demonstrate that YOLO-RDD outperformed YOLO-MobileNet and YOLOv7-tiny in detecting pavement distress. These findings suggest that YOLO-RDD is better equipped to capture detailed features and perform accurate road distress detection.

## 5. Conclusions

This study compares and analyzes six state-of-the-art lightweight models and their target detection capabilities in UAV road space survey scenarios. The main conclusions and contributions are as follows:

- YOLO-MobileNet and YOLO-RDD models based on UAV detection image data were constructed;
- The performances of the six models including Faster R-CNN, YOLOX-s, YOLOv5s, YOLOv7-tiny, YOLO-MobileNet, and YOLO-RDD were compared, and YOLO-RDD has the best overall performance;
- Separate convolution has the strongest lightweight ability, but the accuracy needs to be improved.

The limitation of this paper is that only a few types of distress were included, which is due to the publicly available dataset. This limitation means the model cannot fully meet the demand of highway survey needs. 

In future studies, problems with roadway facilities such as damage to slopes and retaining walls can be included, severe subsidence, cracks, and potholes can be used as a detection category for pavement distress, and minor cracks can be excluded from the crack category.

## Figures and Tables

**Table 1:** A summary of the related research.

Source	Number of Distress Types	Algorithm Type	Data Type/Collection Method	*mAP*	*Param* (M)
Lei et al., 2020 [27]	8	YOLOv3 based detection	1024 pixels × 512 pixels, front view color images/BaiduMap API	0.8837	/
Li et al., 2021 [38]	5	CNN based	1628 pixels × 1236 pixels, front view (more tend to top-view) grey images/HD industry camera	0.8594 (IoU ≥ 0.5)	/
Ibragimovet al., 2022 [39]	3	CNN based	1965 × 2000 pixels, top view grey images/HD industry camera	0.666 (IoU ≥ 0.7)	/
Li et al., 2022 [40]	8	YOLOv3 based	2592 × 2048 pixels, front view (more tend to top-view) grey images	0.7869 (IoU ≥ 0.5)	/
Wang et al., 2023 [41]	5 (including 4 types of cracks)	YOLOv5 based	640 × 640 pixels, top view color images/UAV	0.7653 (IoU ≥ 0.5)	/
Zhang et al., 2022 [3]	4	YOLOv3 based	500 pixels × 500 pixels, top view color images/UAV	0.6875	/
Wan et al., 2022 [42]	4	YOLOv5 based	640 × 640 pixels, front view color images/low-cost camera	0.576 (IoU ≥ 0.5)	19.8
		Faster R-CNN		0.602	28.3
Zhu et al., 2022 [34]	6	YOLOv3 based	7952 × 5304 pixels divided into 512 × 512 pixels blocks, top view color images/UAV	0.566 (IoU ≥ 0.5)	/

**Table 2:** The structure of the benck module in MobileNet V3.

No.	SE	Residual Structure	Activation Function
benck1	√	×	Relu
benck2	×	×	Relu
benck3	×	√	Relu
benck4	√	×	Hard Wish
benck5	√	√	Hard Wish
benck6	√	√	Hard Wish
benck7	√	×	Hard Wish
benck8	√	√	Hard Wish
benck9	√	√	Hard Wish
benck10	√	√	Hard Wish
benck11	√	√	Hard Wish

**Table 3:** Experimental part parameter configuration.

Parameters	Settings
Learning rate	0.01
Weight Decay	0.1
Learning Momentum	0.937
Batch size	8
Training rounds	200

**Table 4:** Comparative experimental results of the models for the test dataset.

Model	*Param* (M)	*FLOPs* (G)	*mAP@0.5*	*BA*	*F1*
Faster R-CNN	41.3	251.4	0.63	0.588	0.61
YOLOX-s	9.0	26.8	0.67	0.528	0.66
YOLOv5s	7.03	16.0	0.659	0.536	0.65
YOLOv7-tiny	6.03	13.1	0.686	0.534	0.68
YOLO-MobileNet	4.48	6.7	0.460	0.481	0.48
YOLO-RDD	6.02	1.9	0.701	0.553	0.69

## Data Availability

The raw data supporting the conclusions of this article will be made available by the authors upon request.
